# Biomechanical Comparison of Modified Suture Bridge Using Rip-Stop versus Traditional Suture Bridge for Rotator Cuff Repair

**DOI:** 10.1155/2016/9872643

**Published:** 2016-11-15

**Authors:** ZiYing Wu, Chong Zhang, Peng Zhang, TianWu Chen, ShiYi Chen, JiWu Chen

**Affiliations:** ^1^Department of Sports Medicine, Huashan Hospital, Fudan University, Shanghai, China; ^2^Department of Orthopaedics, General Hospital of Pingdingshan Coal Industry Group, Pingdingshan City, Henan Province, China; ^3^Department of Orthopaedics, The Second Affiliated Hospital of Soochow University, Suzhou, China

## Abstract

*Purpose*. To compare the biomechanical properties of 3 suture-bridge techniques for rotator cuff repair.* Methods*. Twelve pair-matched fresh-frozen shoulder specimens were randomized to 3 groups of different repair types: the medially Knotted Suture Bridge (KSB), the medially Untied Suture Bridge (USB), and the Modified Suture Bridge (MSB). Cyclic loading and load-to-failure test were performed. Parameters of elongation, stiffness, load at failure, and mode of failure were recorded.* Results*. The MSB technique had the significantly greatest load to failure (515.6 ± 78.0 N, *P* = 0.04 for KSB group; *P* < 0.001 for USB group), stiffness (58.0 ± 10.7 N/mm, *P* = 0.005 for KSB group; *P* < 0.001 for USB group), and lowest elongation (1.49 ± 0.39 mm, *P* = 0.009 for KSB group; *P* = 0.001 for USB group) among 3 groups. The KSB repair had significantly higher ultimate load (443.5 ± 65.0 N) than USB repair (363.5 ± 52.3 N, *P* = 0.024). However, there was no statistical difference in stiffness and elongation between KSB and USB technique (*P* = 0.396 for stiffness and *P* = 0.242 for elongation, resp.). The failure mode for all specimens was suture pulling through the cuff tendon.* Conclusions*. Our modified suture bridge technique (MSB) may provide enhanced biomechanical properties when compared with medially knotted or knotless repair.* Clinical Relevance*. Our modified technique may represent a promising alternative in arthroscopic rotator cuff repair.

## 1. Introduction

Suture bridge technique has been widely accepted in rotator cuff repair because of its better biomechanical properties and satisfactory clinical outcomes [1]. More commonly in the fixation configuration, the suture bridge involves tying knots in medial row. Some authors have suggested that the medial knots were of importance in achieving higher fixation strength [2]. Busfield et al. [3] illustrated that tying the medial knots of horizontal mattress stitches decreases gap formation and increases ultimate and yield loads when compared with those untied constructs. Mall et al. [4] performed a systematic review to determine whether or not the tying of medial row sutures would provide more fixation strength. Of 5 studies collected in his review, 4 showed enhanced stability with knot-tying procedure, and only 1 study showed no improvement. For the medial row of tying knot, supporters emphasized the efficacy of this construct in terms of the strength improvement, while on the other side, advocates of the knotless fashion believed that there was actually no difference in the strength or clinical outcomes between the two aforementioned constructs; moreover, they suggested that impingement and irritation by the medial knot might be alleviated within the subacromial space using the knotless configuration.

Recently, various novel fashions such as triple-loaded anchor and rip-stop technique have been applied in rotator cuff repair. According to Barber and Drew's research [5], the triple-loaded suture anchors placed in a single row were more resistant to stretching strength than the double row groups. Rip-stop configuration [6] was reported to improve ultimate failure load when compared with single row construct. However, to the best of our knowledge, no biomechanical study has ever been performed to evaluate the medial row knotless fixation with combination of triple-loaded suture anchor and rip-stop technique for rotator cuff repair.

In this study, we adopted the triple-load anchor and the rip-stop technique in medial row to modify the knotless suture bridge technique. The purpose of this biomechanical research was to compare the biomechanical properties of three different repair techniques. The hypothesis was that our Modified Suture Bridge would show greater resistance to cyclic loading and destructive load-to-failure testing than other two suture bridge configurations.

## 2. Materials and Methods

Twelve pair-matched fresh-frozen human cadaveric shoulders with intact rotator cuff were harvested from 12 donors (10 males and 6 females; mean age of 56 years [range 52–63 years]). All specimens were stored at −20°C until thawed at room temperature 5 h prior to the application. No specimen had evident pathologic conditions such as rotator cuff tears or history of previous shoulder surgery. All soft tissues were removed from the shoulder. The supraspinatus tendon was reattached to humeral footprint area after being sharply detached from the infraspinatus tendon posteriorly and the rotator interval anteriorly.

## 3. Surgical Procedure

In our study, all constructs adopted anchors in identical number (two medial and two lateral) and varied types. For the anchors, in the medial side, we used the Bio-Corkscrew FT 5.5 double loaded with No. 2 FiberWire (Arthrex, Naples, FL, USA) or Healix PEEK™ 5.5 triple loaded with ORTHOCORD® #2 sutures (DePuy Mitek, Inc., Norwood, MA, USA); in the lateral side, two 3.5-mm PushLock Anchors (Arthrex, Naples, FL) were applied as fixation devices in all 3 groups. Although various suture materials were adopted (Orthocord and FiberWire), they were equivalent in load-to-failure strengths [7, 8].

The 2 medial row anchors were inserted just along the articular margin of humeral head in a 45° angle [9] with the distance of 12.5 mm between two anchors in anteroposterior direction [10].

The anterior anchor in medial row was placed 5 mm posterior to the bicipital groove. The 2 lateral row anchors were inserted at a perpendicular angle to the cortical surface of the humerus, 10 mm distal to the lateral margin of footprint [11]. All the specimens were randomly divided into one of three experimental groups, with 8 specimens per group, for rotator cuff repairs.

### 3.1. The Medially Knotted Suture Bridge (KSB)

**Figure 1:**
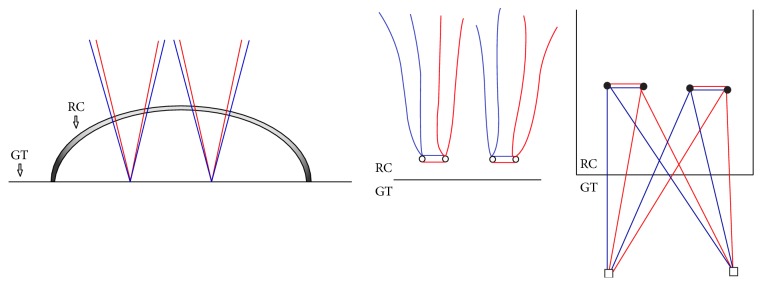
The medially Knotted Suture Bridge (KSB) technique. Black solid circle means knot. RC, rotator cuff; GT, greater tubercle.

This technique utilized two medial 5.5 mm Bio-Corkscrew FT Anchors (Arthrex, Naples, FL) with two No. 2 FiberWire sutures (Arthrex, Napes FL) from each anchor. Two different limbs, one from each suture in the same anchor, perforated through the cuff tendon simultaneously (in a single pass) [5]. Then repeatedly, the remaining two different limbs were passed through tissue 4 mm apart horizontally. Two perforations were centered about anchor. Corresponding suture limbs from the same suture were tied in horizontal mattress configuration using a sliding 3 half-hitch knot followed by 3 alternating half-hitches. Each lateral anchor received two different strands from each medial anchor in a crossing pattern. The construct consisted of 4 penetrations through the cuff tissue. The sutures were placed 15 mm medial to the free edge of the tendon (Figure 1).

### 3.2. The Medially Untied Suture Bridge (USB)

**Figure 2:**
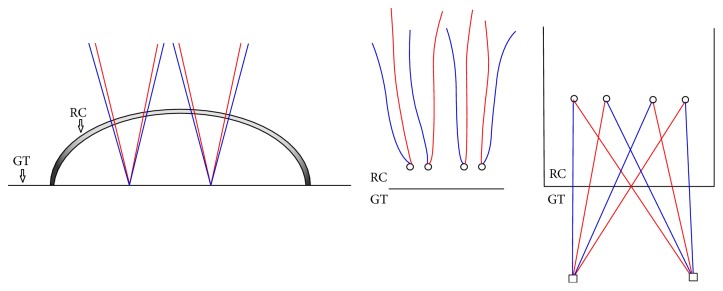
The medially Untied Suture Bridge (USB) technique. Black hollow circle means knotless. RC, rotator cuff; GT, greater tubercle.

It was similar to the KSB technique except for the fact that the sutures from the medial row anchors were untied and simply passed across over rotator cuff tendon (Figure 2).

### 3.3. The Modified Suture Bridge (MSB)

**Figure 3:**
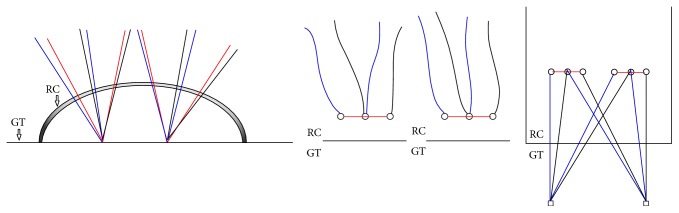
The Modified Suture Bridge (MSB) repair.

This configuration used two triple-loaded anchors in medial row and combined rip-stop technique and knotless repair. Three tendon perforations with respective 3-mm interval for 6 strands of each anchor were performed. Two different limbs, one from each suture in the same anchor, perforated through the cuff tendon simultaneously (in a single pass) in a parallel fashion. The same procedure was performed for the remaining 4 limbs of the same anchor. Two tails of a suture from the same anchor through 1st and 3rd perforation were tied to create a mattress stitch and to serve as a rip-stop stitch. The remaining 4 tails from each medial anchor were not tied and passed laterally to create a crossing pattern over rip-stop stitch and the rotator cuff, secured with two lateral row anchors. The construct consisted of 6 penetrations through the cuff tissue (Figure 3).

## 4. Biomechanical Testing

Biomechanical testing was performed with an Instron Materials Testing Machine (Instron model 1321; Instron, Canton, MA). The humerus was cut in the mid-shaft region 10 cm distal to the surgical neck. This fixation method was similar with the previous study [12].

**Figure 4:**
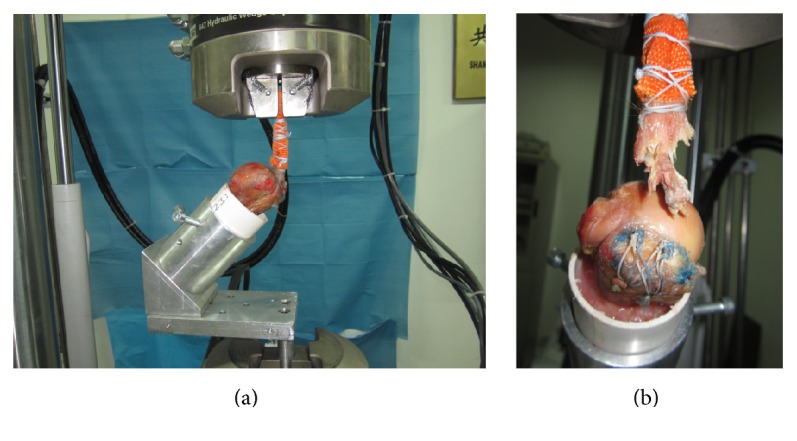
(a) The fixation method of specimens was shown in the image with use of a custom soft-tissue clamp proximally and multiple fixations distally. (b) The construct failed because of suture pulling through the cuff tendon.

The humeral shaft was potted into PVC tubing using plaster. The PVC tube was placed into metal pipe that was mounted to the materials testing machine. Two large screws were perpendicularly placed into metal and PVC tube to further secure the humeral shaft clamped in a custom-made fixation device with the supraspinatus tendon pulled at 45° to the humeral shaft to mimic the physiologic pulling of the tendon as previously described [13, 14]. The medial end of the supraspinatus tendon was wrapped with a cotton bandage and underwent cross-stitches using W4843 Ethibond Excel suture (Ethicon, Johnson & Johnson, Brussels, Belgium) in order to increase friction and eliminate rotator cuff slippage. Subsequently, the medial free end of the supraspinatus tendon was secured with a custom soft-tissue clamp at the proximal end of the materials testing machine. The experimental setup for mechanical testing is shown in Figure 4(a). All specimens were kept moist with 0.9% saline solution at 22°C room temperature during preparation and testing.

The specimens were pretensioned to 10 N for 60 seconds and then loaded cyclically from 10 to 180 N at 1 Hz for 200 cycles and finally loaded until failure occurred at 33 mm/s. This biomechanical testing protocol was similar with the previous study [15, 16]. A video digitizing system (MATFOLT 2D-DIC, Shanghai) was used to measure elongation throughout testing.

### 4.1. Loading Evaluation

Elongation was defined as the difference of the load cell displacement between cycle 200th and 1st during cyclic test, as previously described [15]. The definition of ultimate failure was the peak strength. Stiffness was calculated from the slope of the linear portion of the load-displacement curve. Modes and sites of failure were documented. The study was approved by the ethics committee of Fudan University and performed in accordance with the Declaration of Helsinki.

### 4.2. Statistical Analysis

Based on our preliminary results and previously published data [17], the load at failure was assumed to be 60 N with a standard deviation of 40 N. In order to detect a difference with a power of 0.8 and a significance level of 0.05, eight specimens for each repair group were needed. Prior to the test for difference between groups, Shapiro-Wilk test for normal distribution and the test of equal variance was performed. According to the normality test and the test for homogeneity of variances, one-way ANOVA, followed by post hoc LSD test (Fisher's least significant difference) in case of significance, was performed for data of load to failure and elongation, while the Kruskal-Wallis test was used to detect any difference between groups in terms of stiffness. And *P* < 0.05 was considered significant. Statistical analysis was performed with SPSS for Windows, version 11.5 (SPSS, Inc., Chicago, IL, USA).

## 5. Results

Table 1 summarizes the results of cyclic and load-to-failure testing. The MSB technique exhibited the significantly greatest load to failure (515.6 ± 78.0 N, *P* = 0.04 for KSB group; *P* < 0.001 for USB group), stiffness (58.0 ± 10.7 N/mm, *P* = 0.005 for KSB group; *P* < 0.001 for USB group), and lowest elongation (1.49 ± 0.39 mm, *P* = 0.009 for KSB group; *P* = 0.001 for USB group) among 3 groups. For the remaining groups, the KSB repair had significantly higher ultimate load (443.5 ± 65.0 N) than USB repair (363.5 ± 52.3 N, *P* = 0.024). The stiffness of the construct was 39.0 ± 6.0 N/mm in KSB group and 34.63 ± 7.3 N/mm in USB group. The elongation was 2.29 ± 0.57 mm in KSB group and 2.63 ± 0.68 mm in USB group. There was no statistical difference in stiffness and elongation between KSB and USB technique (*P* = 0.396 for stiffness and *P* = 0.242 for elongation, resp.). The failure mode was suture pulling through the cuff tendon in all specimens (Figure 4(b)). No anchor pulling out of bone or suture slipping out of anchor was observed.

## 6. Discussion

In this study, the Modified Suture Bridge repair using rip-stop technique and triple-loaded anchor was compared with the other two suture bridge techniques with or without knots in medial row. The present outcome revealed the statistical improvement of biomechanical properties in the modification fashion, which confirmed our initial hypothesis.

Suture bridge technique has been achieving popularity in rotator cuff repair for the last few years [18]. When compared with double row technique, suture bridge repair has been thought to lead to improved contact area [19, 20], increased yield load [21], and reduced operative time. Additionally, more strength and contact area could lead to better healing [22].

Regarding the conventional suture bridge technique, the medial row knot was tied by using horizontal mattress configuration [1]. Busfield et al. [3] confirmed the importance of the medial knots in protecting the repair site from biomechanical stresses because of increase in gapping at the repair site and decrease in load transmission. Furthermore, they found that the lack of medial knots resulted in the altered biomechanical characteristics, involving greater gapping and decreased load at failure. Data from the current study also revealed the load at failure of cadaveric supraspinatus tendon reconstruction to be significantly greater in KSB group than that in USB group.

However, the knots may loosen throughout repetitive load, causing gap formation and inhibiting the tendon-bone healing [19, 23]. Moreover, knots in medial row could cause knot irritation or impingement with acromion [24]. According to the analysis by Hug et al. [25], knots in medial row could result in disadvantageous effect on medial row of suture bridge technique. Mazzocca et al. [26] found that the tied knots could lead to a strangulation of the rotator cuff tendon at the medial row and resulted in failure of the medial row during cyclic loading. Furthermore, Yamakado et al. [27] reported that knots were caught between the cuff and the greater tuberosity in several patients of medial row failure after double row reconstruction. However, the medial row construct played a critical role in double row repair because the load transmission from tendon to bone initiated medially [28]. Research focusing on double row rotator cuff repair had suggested that the medial row contributed most to the overall strength with clinical follow-up literature for suture bridge repair with magnetic resonance image showing that the most common retear tended to occur at the medial row [29, 30].

Recently, knotless repairs have been introduced as a refined technique [31, 32]. The advantages of knotless technique included reducing surgery time, eliminating medial and lateral knot impingement. It might also reduce medial strangulation and perforation of the rotator cuff tendon because of missing medial mattress stitches and alleviate irritation of the medial knot within the subacromial space [25, 33]. Rhee et al. [34] performed knotless suture bridge technique for patients with full-thickness rotator cuff tears. When compared with conventional knot-tying suture bridge technique, they found a significant lower retear rate for knotless technique. Furthermore, there was no medial cuff failure in the knotless group while 72.7% retear occurred at the musculotendinous junction for the conventional knot-tying group. This result confirmed the lower frequency of the medial cuff failure in knotless reconstruction techniques [25]. In an effort to acquire preservation of advantages of knotless repair, our modified technique applied knotless technique in medial row except knots in rip-stop technique. However, knotless technique presented inferior initial fixation strength in ultimate load when compared with knot-tying reconstruction, which was consistent with the results of our current study.

Rip-stop suture configurations [6] have been shown to improve load to failure compared with simple or mattress stitch patterns and to provide resistance to tissue cutout. The rip-stop technique strengthened the medial row resistance by distributing the medial-to-lateral tensile strengths. Furthermore, Burkhart et al. [6] confirmed that ultimate failure load of the load-sharing rip-stop construct for rotator cuff repair was 1.7 times the ultimate failure load of a single row construct in cadaveric models.

Triple-loaded anchor has also been proved to possess superior tensile load and ultimate tensile strength in single row when compared with double row technique. The addition of one more suture to a single anchor maximized the strength of a suture anchor construct by significantly increasing the tissue-holding strength over that of 2 sutures [35, 36]. Lorbach et al. [37] reported that the single row repair using triple-loaded anchors and modified suture configuration was similar to the double row suture bridge technique in load to failure and cyclic displacement irrespective of the tested initial sizes of the rupture. However, the tested single row repair consistently restored a less footprint area than the double row method. They further suggested that single row repairs with modified suture configurations might simultaneously merit the superior biomechanical properties and the footprint coverage enhancement that were advocated in double row repairs, moreover, in a satisfactory cost performance by avoiding the high costs of the double row technique. Additional fixation points with use of triple-loaded anchors enhanced the biomechanical strength of repair construct. To improve initial fixation strength, stiffness, and gap formation in knotless technique, we combined the rip-stop technique with triple-loaded anchor for medial row fixation in this research.

Differing from Burkhart's technique in which the rip-stop suture was independently secured, in our technique the modified rip-stop suture was based off the same anchor triple-loaded anchor, and other suture limbs were secured in lateral row fixation without knot tying.

To the best of our knowledge, there is currently no biomechanical study on the combined application of triple-loaded anchor and rip-stop technique in medal-row configuration of suture bridge repair. To reduce knot irritation, impingement, and improve initial weak fixation strength in knotless repair, we have designed the modified rip-stop fixation in the present study to determine whether or not this method of reconstruction could provide better biomechanical properties than the other two groups. Data from the current study demonstrated that our modification method eliminated the need for suture knots medially and had statistically greater elongation, stiffness, and ultimate load when compared with traditional knot-tying suture bridge fixation.

We believe that knots of medial row in conventional suture bridge technique not only hindered load transmission from medial row to lateral row as well as the load distribution but also caused more strength borne by medial rotator cuff tendon. Moreover, we also believed that knotless repair could maintain the load transmission and rip-stop repair could improve load distribution, whose union enhances resistance to pullout. In this research, our Modified Suture Bridge (MSB) technique may provide greater biomechanical properties when compared with medially knotted or knotless repair.

Early motion following rotator cuff repair has been reported to be beneficial to prevent postoperative stiffness and muscle atrophy [38, 39]. Düzgün and coauthors theorized that active mobilization could prevent the negative effects of immobilization and promote the rapid recovery of daily activities [40]. Nevertheless, early postoperative motion may have strain on the repaired rotator cuff and detrimental effects on tendon-bone healing for rotator cuff repair [41] and even result in anatomic healing failure [42, 43]. However, an ideal rehabilitation program, beneficial to tendon-to-bone healing while preventing shoulder postoperative stiffness, has not been definitively established [44]. Gerber et al. theorized that the “ideal repair should have high initial fixation strength, allow minimal gap formation, and maintain mechanical stability until solid healing” [45]. Superior construct in biomechanics could provide a lower retear rate and improved tendon-bone healing when early rehabilitation is performed [46]. To allow for early postoperative rehabilitation while keeping repair integrity, the key is to achieve excellent biomechanical properties [20]. In an randomized controlled trial, Franceschi et al. reported lower retear for double row repair of rotator cuff tear in selected patients at a high risk of shoulder stiffness compared with single row repair after an accelerated rehabilitation protocol [46]. This shows that superior fixation strength could evidently improve tendon-bone healing. However, our results suggest that our modified knotless suture bridge technique may be a better surgical method because it allows the repair to withstand more strength and thus may better support early postoperative rehabilitation programs.

There were some limitations in this study that might compromise the clinical implications of our research. First, the experimental model was produced with fresh-frozen cadaveric tendons and sharp tenotomy rather than the degenerative tendon tissue relevant to a clinical condition. Thus, the biomechanical data in our study perhaps overestimated the initial strength of a primary tendon reconstruction. Secondly, the different suture anchor and materials used in our study might influence our study outcomes. However, they had equivalent load-to-failure strengths. Thirdly, this biomechanical study investigated only primary strength immediately following surgical reconstruction and could not assess the effects of biological factors such as tendon healing. Nonetheless, tendons could retear shortly after surgery: Huijsmans et al. [47] reported that 55% of retears occurred within 3 weeks. Moreover, we did assess primary load-bearing ability during another important phase, the early rehabilitation period. Further investigations were still necessitated to evaluate and compare biomechanical properties after biological tendon healing and clinical outcomes of the three surgical techniques in humans.

## 7. Conclusion

Our modified technique showed achievements of better biomechanical properties including statistical increases in elongation, stiffness, and ultimate load. These results, furthermore, proved that our modification could both maintain advantages of knotless technique in medial row and provide enhanced biomechanical characteristics when compared with knot-tying repair. Our modification might present a promising alternative in arthroscopic rotator cuff repair.

## Figures and Tables

**Table 1:** Biomechanical comparison of 3 constructs.

	KSB	USB	MSB
Load to failure, N	443.5 ± 65.0^&^	363.5 ± 52.3	515.6 ± 78.0^*∗*^
Stiffness, N/mm	39.0 ± 6.0	34.63 ± 7.3	58.0 ± 10.7^#^
Elongation, mm	2.29 ± 0.57	2.63 ± 0.68	1.49 ± 0.39^$^

*∗*: MSB group had significantly higher value compared to that of KSB group and USB group (*P* = 0.04 for KSB group; *P* < 0.001 for USB group).

#: MSB group had significantly higher value compared to that of KSB group and USB group (*P* = 0.005 for KSB group; *P* < 0.001 for USB group).

$: MSB group had significantly higher value compared to that of KSB group and USB group (*P* = 0.009 for KSB group; *P* = 0.001 for USB group).

&: KSB group had significantly higher value compared to that of USB group (*P* = 0.024).
